# Response of lymphocyte subsets and cytokines to Shenyang prescription in Sprague-Dawley rats with tongue squamous cell carcinomas induced by 4NQO

**DOI:** 10.1186/1471-2407-7-40

**Published:** 2007-03-05

**Authors:** Canhua Jiang, Dongxia Ye, Weiliu Qiu, Xiuli Zhang, Zhiyuan Zhang, Di He, Ping Zhang, Wantao Chen

**Affiliations:** 1 Department of Oral & Maxillofacial Surgery, Ninth People's Hospital, School of Medicine, Shanghai Jiao Tong University, Shanghai Key Laboratory of Stomatology, Shanghai 200011, China; 2 Department of Oral Clinical Immunology, School of Stomatology, Shanghai Jiao Tong University, Shanghai 200011, China; 3 Department of Oral & Maxillofacial Surgery, Xiang Ya Hospital, Xiang Ya Medical College, Central South University, Changsha, Hunan 410008, China

## Abstract

**Background:**

The study was designed to investigate immunocompetence in relation to cancer progression in rat and to assess the effect of the traditional Chinese anti-cancer medicine, "Shenyang" prescription, on immunity.

**Methods:**

4-Nitroquinoline-1-oxide (4NQO) was administered to 80 Sprague-Dawley (SD) rats via the drinking water for up to 36 weeks. Tongue squamous cell carcinoma (SCC) was confirmed by pathological examination in 61 rats. "Shenyang" prescription was administered to subgroups of these rats, and blood samples were taken before and after treatment. Lymphocyte subsets were determined by flow cytometry. Serum Th1 and Th2-type cytokines were assessed by an enzyme-linked immunosorbent assay.

**Results:**

As the cancer progressed at the tongue root, the percentage of CD3+CD4+ T lymphocytes and NK cells and the levels of IFN-γ and IL-2 decreased gradually, while the percentage of CD3+CD8+ T lymphocytes and the levels of IL-4 and IL-10 increased. The CD4+/CD8+ ratios were lower in the cancer groups than in the control group. However, after administering "Shenyang" prescription, the levels of CD3+CD4+ T lymphocytes, NK cells, IFN-γ and IL-2 increased, while the CD3+CD8+ T lymphocyte counts and the levels of IL-4 and IL-10 decreased.

**Conclusion:**

4NQO-induced lesions were good models for exploring oral cavity carcinogenesis. The rats with 4NQO-induced SCC demonstrated abnormalities in lymphocyte subsets and a shift from Th1-type to Th2-type, which were good models for assessing the effect of anticancer agent on immunity. Oral cancer progression was associated with an aggressive disturbance of immune function. "Shenyang" prescription has the ability to improve the disturbance of immune function.

## Background

Oral cancer is a major global public health problem, causing high morbidity and mortality that have not improved in decades [1]. Squamous cell carcinomas (SCCs) are the most common type of oral cancer. Although new operative techniques and adjuvant measures such as chemotherapy and radiotherapy against oral SCCs have progressed, patients with advanced oral SCCs still have a poor prognosis, with a 5-year survival rate of 65% [2]. Among patients with early stage cancers (I and II), about 30% cannot be cured, while for patients with advanced stage tumors (III and IV) this percentage may be as high as 70% [3]. To develop new methods and improve existing protocols for diagnosis and treatment, an animal model that mimics the natural course of SCCs was mandatory. Application of 4-nitroquinoline-1-oxide (4NQO) to rat tongues is a reliable procedure for inducing oral SCCs [4]. 4NQO, a water-soluble quinoline derivative, has been extensively investigated and is known to cause the formation of DNA adducts. In bacteria, 4NQO induces base-pair changes (GC→AT) and deletion mutations. This mutagenic action requires the conversion of 4NQO to 4-hydroxyaminoquinoline-1-oxide, which binds covalently to nucleic acids and damages chromosomes [5,6]. In eukaryotic cells, 4NQO induces diplochromosomes, indicative of disturbed chromosome replication. 4NQO is a powerful carcinogen in several organs, and it can specifically induce tongue SCC when applied in low concentrations via drinking water. The resulting sequential changes and morphological features resemble those seen during the progression of human tongue squamous cell malignancy. Therefore, 4NQO-induced rat tongue SCC is an excellent model for studying early events in oral carcinogenesis.

Our previous clinical research showed that "Shenyang", a traditional Chinese medicine prescription made mainly from *Radix codonopsitis, Radix astragali, Salvia miltiorrhiza *and *Glossy privet fruit*, increases the post-operative survival rate of patients with oral SCCs and the mechanism of action may involve stimulation of the patient's immune function [7]. The main active constituents in *Radix codonopsitis *are tangshenoside and atractylenolide, astragalus glucans and flavonoids in *Radix astragali*, tanshinones and cryptotanshinone in *Salvia miltiorrhiza*, nuzhenide and olenropein in *Glossy privet fruit*. Their pharmacological actions include effect on immune function, enhancement in SOD activity, increase in white cell count, liver protection, and hypoxia tolerance. There is compelling evidence that immune responses are impaired in patients with oral SCCs. Some studies have shown that a growing tumor burden correlates with aggravating changes in immunity. Numerous factors are involved in regulating host immunity. Of critical importance are the absolute and relative proportions of lymphocytes and NK cells, and the functional balance between T-helper type 1 (Th1) and type 2 (Th2) lymphocytes. However, little is known about the relationship between immunocompetence and the growth of tongue squamous cell carcinoma induced by 4NQO. Recent views of immunity encouraged us to perform a multiparametric immunological assessment of a rat tongue cancer model, to evaluate its relationship to tumor progression and immune function, and to observe the effect of "Shenyang" prescription on immune function.

## Methods

### Animals and application of 4NQO

4NQO was obtained as a powder (Sigma, USA) and dissolved in water to a final concentration of 0.02 g/l. This low concentration of 4NQO consistently induced oral SCC in earlier rodent studies [8,9]. Eighty Sprague-Dawley (SD) male rats, aged 6–7 weeks, were fed 4NQO in their drinking water for 36 weeks. During induction, two rats were sacrificed under general anesthesia at the 6th, 12th, 18th, 24th and 30th weeks for pathological examination. Another 20 SD rats received pure water (control group). Experimental Animal Administrative Committee of Shanghai granted ethical approval for the animal experiments.

### Determination of immune function in the rat groups

At the end of the 36th week, ten rats had been sacrificed for pathological examination and nine had no tumors or had died during application of the carcinogen. To determine the relationship between immune function and the progress of carcinogenesis induced by 4NQO, abnormalities of immune function were tested in the 61 surviving rats. The oral cavities of these rats were examined under general anesthesia and the animals were divided into three groups according to tumor diameter. Group A comprised 24 rats with tongue mucosal ulcers in which the longest diameter was less than 2 mm and with no palpable tumor invasion; group B comprised 21 rats with invasive tumors in which the longest diameter was less than 5 mm; and group C comprised 16 rats with invasive tumors of diameter greater than 5 mm. The 20 rats in the control group had normal tongue mucosas.

### Treatment and application of "Shenyang" prescription

The 61 rats with oral tumors were divided into 4 groups by stratified random sampling: "Shenyang" prescription group I (16 mice), "Shenyang" prescription group II (15 mice), positive controls (15 mice) and negative controls (15 mice). All the 61 rats had squamous cell carcinoma with different stage and size. The pathological results had been confirmed by three pathologists. Each medicated group has a comparable number of rats with each of the 3 lesions. The "Shenyang" prescription groups I and II received "Shenyang" as granules. "Shenyang" prescription was produced by Shanghai Third Chinese Medicine Factory in China. All herbs (*Radix codonopsitis*, *Radix astragali*, *Salvia miltiorrhiza and Glossy privet fruit*) used in the present study were provided by Shanghai Herbs Company (Shanghai, China). Traditional controls on cultivation and modern technologies on production were used to ensure that the composition (level of active ingredient) was stable for each of the 4 components. The percentages of the 4 components were *Radix codonopsitis *(30%), *Radix astragali *(30%), *Salvia miltiorrhiza *(10%) and *Glossy privet fruit *(30%)[7]. The doses, determined by the conversion of body surface area proportion, were 1026 mg/100 g body weight for group I and 256.5 mg/100 g for group II. The positive controls received 7.6 mg/100 g Acanthopanax senticoside (Shanghai Xin Yi Jia Hua Pharmaceutical Company). The doses for "Shenyang" prescription group II and the positive control were equivalent to human dosages. We observed the average daily water intake of each rat from 2 weeks in order to calculate the concentration of medicine to be administered. The medicine was then dissolved into water at the appropriate concentration and administered for 15 days. The negative controls received distilled water.

### Blood sampling

Before and after treatment, femoral vein blood samples were obtained in sterile heparinized tubes from all rats under general anesthesia with methoxyflurane vapor. The serum samples were collected by centrifugation, stored at -80°C and thawed immediately before the enzyme-linked immunosorbent assay (ELISA).

### Flow cytometry

T lymphocyte subpopulations were quantified by flow cytometry (FACSCalibur; Becton Dickinson, USA). Samples were prepared by adding 5 μl of fluorescent monoclonal antibodies against CD3, CD4, CD8 and CD161a (Pharmingen, Becton Dickinson, USA) to 100 μl of heparinized whole blood. CD3+CD4+ (T helper cells) and CD3+CD8+ (T suppressor cells) were measured and natural killer (NK) cells were recognized as CD3-CD161a+ [10]. Mouse IgG isotype control antibodies were used to estimate the amount of nonspecific binding. Cell preparations were left on ice in the dark for 15 minutes. Erythrocytes were then lysed once and, after washing the WBC pellet, the tubes were placed on ice in the dark until flow cytometric analysis, which was performed on the same day.

### Cytokine assays

The serum samples were thawed immediately before testing. Supernatant cytokine (IFN-γ, TFN-α, IL-2, IL-4 and IL-10) levels were measured using commercially available ELISA kits (Pharmingen, Becton Dickinson, USA) [11]. Monoclonal antibodies specific for rat IFN-γ, TFN-α, IL-2, IL-4 and IL-10 were pre-coated on to microplates. Standards, controls and samples were pipetted into the wells. After washing to remove unbound materials, enzyme-linked polyclonal antibodies specific for rat IFN-γ, TFN-α, IL-2, IL-4 and IL-10 were added to the wells. After washing, the substrates were added. The enzyme reactions yielded blue products that turned yellow when the stop solutions were added. The absorbance was measured with an ELISA reader (Biorad, USA) at 450 nm, corrected by the absorbance at 630 nm, and a standard curve was used. Samples were assayed in triplicate and the results were expressed as the average of the three absorbance readings. The serum cytokines were considered to be positively expressed when their concentrations were greater than or equal to the detection limits (15 pg/ml).

### Tumors dissection and histological preparation

After phlebotomy, the rats were sacrificed to resect the tongue tumors. The volumes of all the tumors were measured by Vernier calipers. Gross lesions were fixed in 10% buffered formalin, sectioned and stained with hematoxylin and eosin. All slides were reviewed by three pathologists using light microscopes.

### Statistical analysis

All data were analyzed using SAS for Windows 98. Results were expressed as means and standard deviations. Groups were compared by *t*-tests for continuous variables. Statistical significance was defined as *P *< 0.05.

## Results

### Characteristics of SD rat model

To determine whether 4NQO reproducibly caused a spectrum of neoplastic changes in the tongue, 80 SD rats were treated with 4NQO for 36 weeks; 61 rats with tumors survived after this period. Ten were sacrificed for pathological examination during induction. Nine rats had no tumors or had died of pneumonia or gastroenteritis.

**Figure 1:**
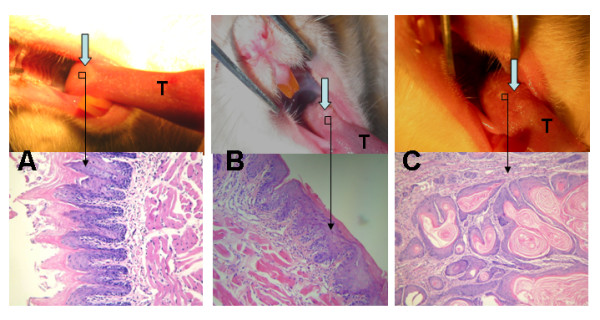
**Both gross and microscopic changes in SD rats' tongues mucosas**. A. Normal mucosa of tongue base at 6^th ^week (gross observation and HE ×200); B. Epithelial dysplasia of tongue base at 24^th ^week (gross observation and HE ×200); C. Squamous cell carcinoma of tongue base at 36^th ^week (gross observation and HE ×200). T represents tongue.

Morphologically, the lesions at the 6th, 12th, 18th, 24th and 30th weeks that grew in response to 4NQO demonstrated a series of squamous cell transformations from normal mucosa (Fig. 1A) through epithelial dysplasia (Fig. 1B) to squamous cell carcinoma (Fig. 1C). Exposure to 4NQO caused both gross and microscopic changes in the tongues. The macroscopic lesions were shown by microscope to be squamous cell carcinomas.

### Cell counts

**Figure 2:**
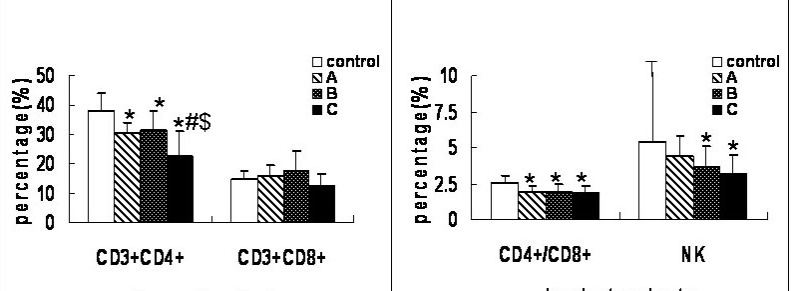
**Alteration of peripheral lymphocyte subsets and NK cells with tumor progression**. T lymphocyte subpopulations were quantitated by flow cytometry. Samples were prepared by adding 5 μl of fluorescent monoclonal antibody of CD3, CD4, CD8 and CD161a to 100 μl of heparinized whole blood. CD3+CD4+ (T helper cell) and CD3+CD8+ (T suppressor cells) were measured and natural killer (NK) was recognized as CD3-CD161a+. Values (except for CD4+/CD8+) represent percentage (%). Data are expressed as averages and *SD*. * means compared with control group, *P *< 0.05. # means compared with group A, *P *< 0.05. $ means compared with group B, *P *< 0.05.

During the progression of the tumors, rats in the experimental and control groups had different T lymphocyte subsets and NK cells counts. The results were summarized in Fig. 2. The numbers of CD3+CD4+ T cells and the CD4+/CD8+ ratios were consistently lower in groups A, B and C than in the control group (*P *< 0.05). The CD3+CD4+ T lymphocyte counts were significantly lower in group C than in groups A or B (*P *< 0.05), but the percentages in groups A and B were comparable (*P *> 0.05). In addition, the NK cell counts were similar in group A and control (*P *> 0.05), but as the lesion enlarged, there were statistically significant differences among groups B, C and control (*P *< 0.05).

**Figure 3:**
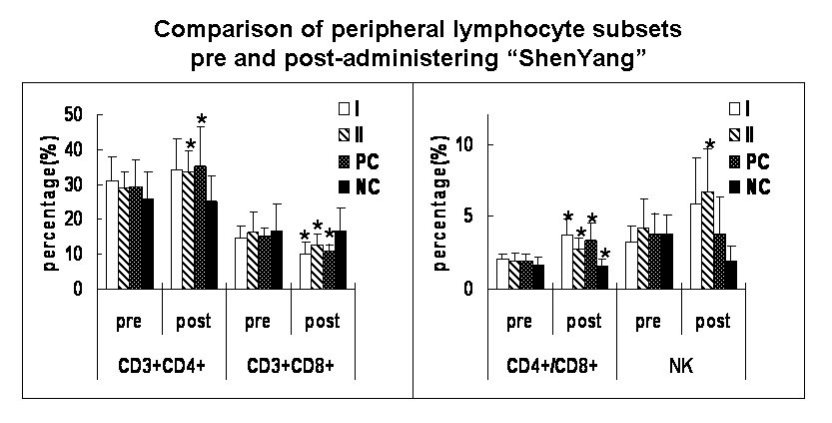
**Comparison of peripheral lymphocyte subsets pre- and post-administering "Shenyang" prescription**. T lymphocyte subpopulations were quantitated by flow cytometry. Samples were prepared by adding 5 μl of fluorescent monoclonal antibody of CD3, CD4, CD8 and CD161a to 100 μl of heparinized whole blood. CD3+CD4+ (T helper cell) and CD3+CD8+ (T suppressor cells) were measured and natural killer (NK) was recognized as CD3-CD161a+. Values (except for CD4+/CD8+) represent percentage (%). Data were expressed as averages and *SD*. * means statistic difference (*P *< 0.05) between pre- and post-administering "Shenyang"; I means "Shenyang" prescription group I; II means "Shenyang" prescription group II; PC means positive control group; NC means negative control group.

Medication produced differences in cells counts, and Fig. 3 shows the results. The percentages of CD3+CD4+ T lymphocytes were significantly higher after medication than before in "Shenyang" prescription group I and II as well as in the positive controls (*P *< 0.05). So were the CD4+/CD8+ ratios in all medicated groups. However, the percentages of CD3+CD8+ T lymphocytes were significantly reduced after medication in all groups except the negative controls (*P *< 0.05). The NK cell counts were significantly increased after medication in "Shenyang" prescription group I and decreased in the negative controls (*P *< 0.05).

### Th1 and Th2 type cytokine production

**Figure 4:**
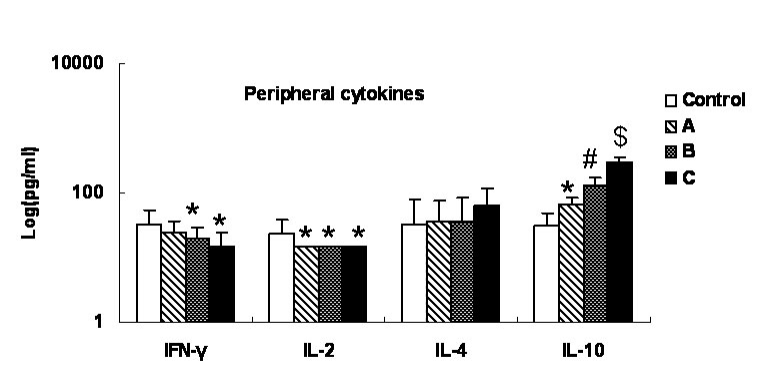
**Alteration of peripheral cytokines with tumor progression**. The serum samples' supernatant cytokines (IFN-γ, TFN-α, IL-2, IL-4 and IL-10) levels were measured with commercially available ELISA kits. Data were expressed as averages and *SD*. * means compared with control group, *P *< 0.05. # means compared with group A, *P *< 0.05. $ means compared with group B, *P *< 0.05.

Fig. 4 summarized the changes in cytokine production as the tumor progressed. Serum IFN-γ decreased gradually and was significantly lower in groups B and C than in the control group (*P *< 0.05). The IL-2 concentration in the control group was 24.13 ± 15.12 pg/ml, but below 15 pg/ml in groups A and C. Hence, there were significant differences between each tumor group and the control group (*P *< 0.05). Although there was a tendency for IL-4 and IL-10 concentrations to increase, the IL-4 values were statistically similar in all groups (*P *> 0.05) while IL-10 production differed significantly in each group (*P *< 0.05). The cytokine levels did not vary significantly among the tumor groups at different stages of development of the lesions.

**Figure 5:**
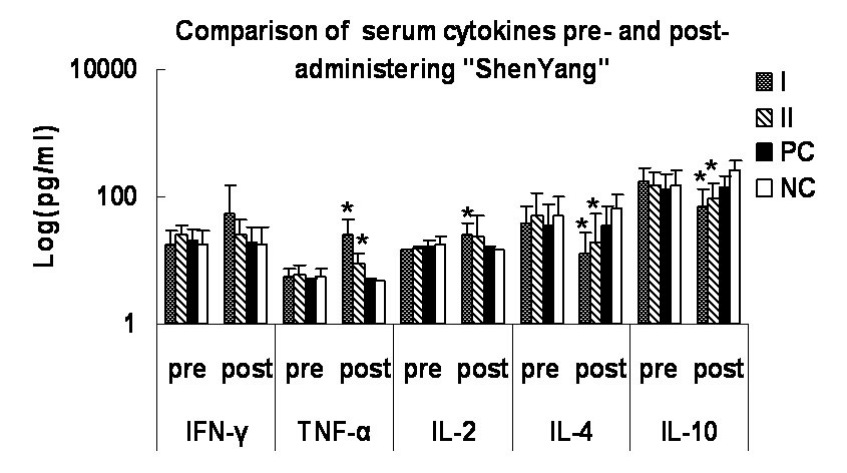
**Comparison of peripheral cytokines pre- and post-administering "Shenyang" prescription**. The serum samples' supernatant cytokines (IFN-γ, TFN-α, IL-2, IL-4 and IL-10) levels were measured with commercially available ELISA kits. Data are expressed as averages and *SD*. * means statistic difference (*P *< 0.05) between pre- and post-administering "Shenyang"; I means "Shenyang" prescription group I; II means "Shenyang" prescription group II; PC means positive control group; NC means negative control group.

The effect of medication was shown in Fig. 5. The serum concentrations of TFN-α and IL-2 increased significantly after medication in "Shenyang" prescription groups I and II (*P *< 0.05). IL-4 decreased significantly in "Shenyang" prescription group I than group II (*P *< 0.05). The IL-10 values were very significantly lower in both "Shenyang" prescription groups after medication (*P *< 0.01), while they increased significantly in the negative controls (*P *< 0.01).

## Discussion

Malignant transformation of the oral mucosa is a complicated multi-step and multi-factorial process that is only partly understood. To develop a new strategy for treating oral SCC, it is important to understand the progression of the tumor. To date, however, relevant human and rodent models have been quite limited in number and characterization remains unsatisfactory. Oral cancer can be induced by topical application of the water-soluble carcinogen 4NQO and the level of DNA damage is directly related to the severity of the histological changes [12]. Tang et al. [13] showed that 4NQO changed the expression patterns of the intermediate filament proteins keratin14 (K14) and K1. K14 was expressed in the epithelial suprabasal layers in addition to the basal layer. Moreover, more bromodeoxyuridine was found in the tongue epithelia. Expression of the cell cycle inhibitor p16 decreased, whereas epidermal growth factor receptor expression increased in the tongue.

4NQO-induced lesions appear to be a good model for oral cavity carcinogenesis [14]. Premalignant and malignant changes following 4NQO application are local and occur in the setting of morphologically normal mucosa. No tumors were observed in the digestive tract (including the stomach, intestine and colon) or in the lungs or livers of the 4-NQO-treated rats. This indicates that the rat model of 4NQO-induced oral carcinogenesis simulates aspects of human oral cavity carcinogenesis.

The major disadvantage of the rodent 4NQO model is the long time required for carcinogenesis. The experimental period is more than 40 weeks. The other disadvantage is the relatively high mortality. In this study, 6 rats died of pneumonia or gastroenteritis. A low concentration (0.02 g/l) of 4NQO was used to induce the oral cancer. The deaths of the rats were not associated with hyperkeratotic changes in the stomach [15] and subsequent malnutrition or cumulative toxicity from 4NQO [16].

The results of this study support the concept that host immunity is implicated in the regulation and progression of malignant disease. Patients bearing SCCs usually show systemic immune suppression. This suppression can be measured as a change in numbers of T lymphocytes and NK cells [17,18]. There are scattered data on lymphocyte subsets and immune responses in rat models with SCCs. One recent report suggested that when 4NQO was applied to BALB/c mouse tongues, the growing tumor affected the immune response around the tumor and systemically [19]. Moreover, measures to improve immunological status may enhance the body's ability to eliminate tumor cells after conventional therapy and improve the survival rate. Clinical research has confirmed that "Shenyang" can improve the survival rate of OSCC patients [7]. To obtain normal control values, 20 healthy SD rats were selected in our study. The results demonstrated significant changes in T lymphocyte subsets and NK cells counts, a decreasing ratio of CD4+ to CD8+ T lymphocytes, down-regulation of the Th1 type immune response and up-regulation of Th2 type lymphocyte function as the tumor progressed, and these changes were reversed by application of "Shenyang".

T lymphocytes are known to be intimately involved in anti-tumor immune responses. T cells can affect tumor cells directly, or can act indirectly via the production of cytokines that amplify cytotoxic T lymphocyte (CTL) responses or activate NK cells and macrophages. Various experimental studies have outlined the critical role of CD4+ and CD8+ T cells and NK cells in mediating anti-tumor immunity [20-23]. In agreement with other reports, we observed overall fluctuating differences in the relative proportion of CD4+ and CD8+ T cells between the abnormal and normal groups. A persistently lower CD4+/CD8+ ratio and proportion of NK (CD161a+) cells has been described in advanced lesions. The present study confirms the decrease in the CD4+/CD8+ ratio with increasing tumor burden. This relationship appeared to result mainly from a decrease in CD4+ T cells and an increase in CD8+ T cells, especially in group B, as the tumor progressed.

Many investigators have noted deficient cell-mediated immunity in advanced cancer patients. The current study emphasizes that the proportions and functions of immunoregulatory cells tend to correlate with the stage of malignancy [24]. We explain the decreasing CD4+/CD8+ ratio as predominantly due to a decrease of CD4+ T cells as the tumor progresses. Watson et al [25] found that in tumor-bearing mice, aberrant antigen presentation preferentially expanded CD8+ T cells (but not CD4+ cells), which failed as effectors of cytotoxicity against tumor cells; instead, they acted to down-regulate further immune responses to tumor-associated antigens.

On the other hand, the results also showed that the numbers of CD4+ T cells and the CD4+/CD8+ ratio increased after application of "Shenyang" and Acanthopanax senticoside(AS), while the numbers of CD8+ cells decreased significantly. This indicates that "Shenyang" and AS counter the disordering of T lymphocyte subsets and improve the body's immunity. We also found that the application of 'Shenyang" increased the number of NK cells, while AS did not. Tumor cells rely on the environment for angiogenesis. The interplay between them depends mainly on cytokine secretions [26-28]. The T-helper cytokine profile has been implicated in coordinating the events that potentially result in protective host immunity. Lathers et al [29] demonstrated aberrant cytokine expression in the plasma of patients with more advanced squamous cell carcinoma of the head and neck. In this present study AS was used positive herb for regulating host immunity. By now some studies reported that AS can regulate the cellular immunity and factor, indicating that AS can be used as an assistant drug to regulate the function of immunity in the patients with cancers [30-32].

Agarwal et al [33] found that the development of oral SCC led to polarization of cytokine expression, which became skewed towards the Th1-like response in early stages, while an increasing tumor load skewed it toward a Th2-like response. Studies to define the balance between Th1 and Th2 immune responses in human cancer are just emerging. Our model simulates this progression. Th1-dominant immunity appears critical for inducing anti-tumor cellular immunity in mice [34]. Compared with the control group, SD rats with advancing lesions had down-regulated Th1-type and up-regulated Th2-type cytokine production, which implies a shift from Th1 to Th2. Others have found a normal Th2-type response, but in contrast to our study, in combination with Th1 deficiency [35], whereas van Sandick et al [36] reported that cancer patients demonstrated a shift in the Th1/Th2 balance in favor of Th1. Our study also showed that "Shenyang" induced an up-regulation of Th1-type and a down-regulation of Th2-type cytokines, which is a possible mechanism for improving cellular immune function. The discrepancies may stem to some extent from the variety of techniques used for cytokine measurement. In this study, the ability of T cells to produce cytokines was assessed by ELISA.

## Conclusion

Our results showed that 4NQO is a suitable carcinogen for inducing squamous cell carcinoma in the tongue of SD rat. SD rats with SCCs had decreased CD4+/CD8+ ratios, which were associated with a greater tumor load. An abundance of suppressive CD8+ T cell may be induced by the tumor itself, or at least help the tumor to enlarge. A shift in the Th1/Th2 balance is caused by down-regulation of Th1-type and up-regulation of Th2-type cytokines. The rats with 4NQO-induced lesions are good models for exploring oral cavity carcinogenesis and for assessing the effect of anticancer agent on immunity. The disturbance of immune function in 4NQO-induced oral cancer rats can be reversed by administering "Shenyang" prescription. These data lead us to propose that immunity against malignant tumors is perturbed as the tumor progresses, and that the anticancer function of "Shenyang" prescription is underpinned by the improvement of immune function.

## Competing interests

The author(s) declare that they have no competing interests.

## Authors' contributions

CHJ carried out the cellular phenotype and animal studies, performed the statistical analysis and drafted the manuscript. DXY carried out the immunoassays, and helped to draft the manuscript. WLQ conceived of the study, participated in its design, provided experimental agents and helped to draft the manuscript. ZYZ conceived of the study and participated in its design and coordination. XLZ participated in animal study. DH and PZ performed the statistical analysis, and helped to draft the manuscript. WTC conceived of the study, carried out the animal studies, and draft the manuscript. All authors read and approved the final manuscript.

## Pre-publication history

The pre-publication history for this paper can be accessed here:
